# Reproductive tract non-viral mono- and co-infections in pregnancy: systematic literature review and meta-analysis

**DOI:** 10.1017/ash.2026.10821

**Published:** 2026-09-08

**Authors:** Gustavo Yano Callado, André Luís Franco Cotia, Maria Celidonio Gutfreund, Luana Perrone Camilo, Patrícia Deffune Celeghini, Romy Schmidt Brock Zacharias, Eduardo Félix Martins Santana, Michael B. Edmond, Alexandre R. Marra

**Affiliations:** 1 Faculdade Israelita de Ciências da Saúde Albert Einstein, Hospital Israelita Albert Einstein, São Paulo, São Paulo, Brazil; 2 Department of Obstetrics, Federal University of Sao Paulo, Sao Paulo, Brazil; 3 Department of Medicine, West Virginia University School of Medicine, Morgantown, WV, USA; 4 Department of Internal Medicine, University of Iowa Health Care, Iowa City, IA, USA; 5 Division of Infectious Diseases & Geographic Medicine, Stanford University, Stanford, CA, USA

## Abstract

**Objective:**

To compare maternal and neonatal outcomes between mono-infections and co-infections of the reproductive tract in pregnancy due to bacteria and *Trichomonas.*

**Methods:**

We conducted searches across MedLine, Cumulative Index to Nursing and Allied Health Literature (CINAHL), Cochrane CENTRAL, Web of Science, Scopus, and Embase. Searches from database inception to August 5, 2024, for studies that compared reproductive tract bacterial and *Trichomonas* mono-infections and co-infections in pregnancy with regards to maternal and/or neonatal outcomes. Two independent investigators analyzed each study. Random-effect models were used to estimate pooled odds ratios (ORs) with 95% confidence intervals (CIs), and heterogeneity was assessed using the *I* ^2^ statistic. The Downs and Black scale was used to assess study quality and risk of bias.

**Results:**

Among 1,436 publications, nine studies met inclusion criteria. Mono-infections and co-infections showed no significant differences in delivery before the 34^th^ week of gestation (OR 0.66, 95% CI 0.36–1.22), gestational hypertension (OR 1.30, 95% CI 0.72–2.33), preterm premature rupture of membranes (OR 0.89, 95% CI 0.50–1.58), or Apgar score < 7 at five minutes (OR 0.53, 95% CI 0.20–1.36). The analysis of birth before the 37^th^ week rate (five studies) showed an advantage for the mono-infections group (OR 0.83, 95% CI 0.78–0.89). Mono-infections showed lower rates of neonatal death (OR 0.83, 95% CI 0.78–0.89) and intensive care unit admission (OR 0.16, 95% CI 0.03–0.86).

**Conclusions:**

Reproductive tract non-viral co-infections may exacerbate adverse neonatal outcomes compared to mono-infections. Future studies are needed to establish a more robust correlation between infections numbers and pregnancy outcomes.

## Introduction

The presence of infectious pathogens within the amniotic fluid or placental circulation has been well documented to adversely affect pregnancy outcomes.^1,2^ Among the myriad of microorganisms capable of causing such complications, certain bacterial reproductive tract infections have garnered particular attention due to their established risk profiles. *Chlamydia trachomatis* and *Neisseria gonorrhoeae*, for instance, can ascend from the cervix to the uterus, leading to preterm labor, low birthweight, and neonatal infections.^3,4^

In addition to sexually transmitted infections, other bacteria, including *Ureaplasma spp.* and *Mycoplasma spp.*, may contribute to adverse pregnancy outcomes. These microorganisms, often asymptomatic, can be found in the vaginal flora and have been implicated in various pregnancy complications.^2^

Genitourinary tract infections, such as chlamydia and gonorrhea, generally do not manifest systemically, unlike syphilis, which, being a systemic infection, is inherently associated with a higher risk of preterm labor or fetal death depending on the stage of maternal disease.^5^

A noteworthy observation is the relatively high prevalence of multiple bacterial co-infections among women of childbearing age.^3,6,7^ This phenomenon underscores the importance of investigating the potential synergistic effects of these co-infections on maternal and fetal health.

The aim of this systematic review and meta-analysis was to compare maternal and neonatal outcomes between reproductive tract non-viral mono-infections and co-infections in pregnancy.

## Methods

This systematic literature review followed the Preferred Reporting Items for Systematic Reviews and Meta-Analysis (PRISMA) statement^8^ and the Meta-analysis of Observational Studies in Epidemiology (MOOSE) guidelines.^9^ It was registered in the International Prospective Register of Systematic Reviews platform (PROSPERO registration number CRD42024572051, August 2, 2024) before conducting the literature search.

### Search strategy

A health sciences librarian assisted in the formulation of our search strategy. We executed comprehensive database searches using Medline (PubMed), Cumulative Index to Nursing and Allied Health Literature (CINAHL), Cochrane CENTRAL, Web of Science, Scopus, and Embase. The search scope extended from the initial records in each database to August 5, 2024. The complete search strategy is detailed in the Supplemental Material.

### Inclusion and exclusion criteria

Studies were included if they compared maternal and/or neonatal outcomes for reproductive tract bacterial, *Trichomonas*, or fungal (eg, candidiasis) mono-infections and co-infections in pregnancy. We excluded reviews, non-comparative studies, case reports, case series, commentaries, and opinion papers. Studies that compared bacterial infections with viral infections were also excluded. However, if viral infections were a described in the demographic characteristics the studies were not excluded.

**Figure 1.**
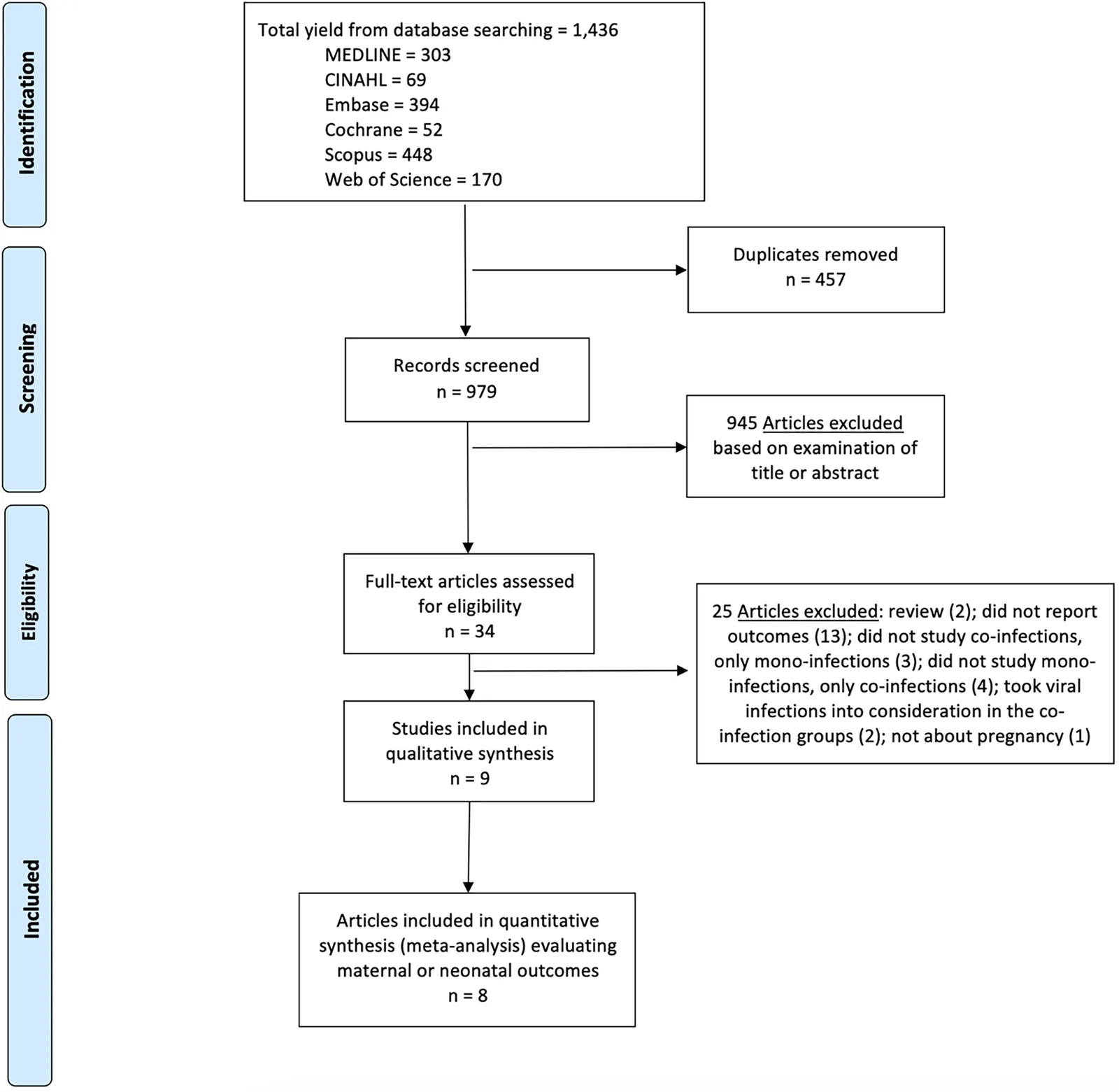
Figure 1 long description. Literature search for articles that evaluated the impact of non-viral reproductive tract mono-infections and co-infections in maternal and neonatal outcomes. CINAHL, Cumulative Index to Nursing and Allied Health Literature.

Two reviewers (G.Y.C., M.C.G.) independently evaluated all titles and/or abstracts, eliminating those that did not meet the inclusion criteria. Discrepancies were addressed through discussion. Following this initial assessment, all remaining studies were fully read, and those that met the eligibility criteria were incorporated into the systematic literature review (Figure 1).

### Data extraction and quality assessment

Of the five independent reviewers (G.Y.C., A.L.F.C., M.C.G., L.P.C., P.D.C.), two independently abstracted data from each article using a standardized abstraction form (Supplemental Form 1). We recorded the publication year, study period, study design, comparison groups, maternal and fetal characteristics, how infections were diagnosed, treatment, maternal and neonatal/pediatric outcomes preestablished in the study protocol, and the main conclusions of each study.

To evaluate the quality of the included studies, we utilized the Downs and Black scale.^10^ Each article underwent an assessment encompassing all original scale questions, resulting in a cumulative score. Question #27 was modified by converting multiple-choice options into a binary yes/no format. The maximum attainable score on this scale was 28. The score for each article was subsequently classified as “good quality” (≥18 points), “fair quality” (14–17 points), or “low quality” (<14 points). Two of the five reviewers independently evaluated the quality of individual components within each article, and any disagreements were resolved through consensus.

### Outcome measures

This systematic literature review explored both maternal and neonatal outcomes. Maternal outcomes included birth before the completion of the 34^th^ and 37^th^ weeks of gestation, gestational hypertension, eclampsia, and preterm premature rupture of membranes (PPROM). Outcomes related to the offspring included spontaneous abortion/stillbirth, neonatal death, sepsis, birthweight, Apgar score < 7 at five minutes, intensive care unit admission, and chorioamnionitis or intra-amniotic infection. All results reported in the included studies, even those not described in our initial protocol, were incorporated into the review.

### Statistical analysis

We conducted a meta-analysis on outcomes reported by at least two studies. Each outcome was individually analyzed using random-effects models to compare non-viral mono-infection and co-infection, calculating pooled odds ratios (ORs) with 95% confidence intervals (CIs). To estimate between-study variance using random effects, we employed the DerSimonian and Laird approach for weight determination.^11^ If an analysis contained a single study representing more than 95% of the analysis weight, we performed a sensitivity analysis excluding the specific study. The first analysis considered all mono-infections and all co-infections, and the second analysis intended to compare specific infections.

We evaluated between-study heterogeneity using both the *I* ^2^ statistic and the Cochran’s Q test. We intended to explore statistical heterogeneity and identify its potential sources, by conducting stratified analyses for each outcome with an *I* ^2^ > 40%, considering factors such as publication date, location, study design, diagnosis, treatment, birthweight, and gestational age (GA). Publication bias was assessed using funnel plots and Egger’s test, with *P* value > .05 indicating no evidence of publication bias. The analyses were executed using R software version 4.1.0 and the Cochrane Review Manager (RevMan) Web edition 7.12.0.

## Results

### Characteristics of the included studies

The initial search resulted in 1,436 articles, of which 457 were duplicates. Of the remaining 979 articles, 34 proceeded to full-text review and, of those, nine met the inclusion criteria.^3,12–19^ The PRISMA flowchart of the study selection is shown in Figure 1. Of the nine included studies, six were retrospective cohort studies,^3,14,16–19^ one was a prospective cohort study,^15^ one was a case–control study,^12^ and one was a cross-sectional study.^13^ Three were conducted in the United States,^13,16,17^ three in East Asia,^14,18,19^ one was a multicenter study with most patients in South America,^3^ one in the Middle East,^12^ and one in Oceania.^15^

According to the inclusion criteria, all studies divided their patients into at least two groups: mono-infection and co-infection. Three studies focused on bacterial sexually transmitted infections, including chlamydia, gonorrhea, and syphilis.^3,13,16^ Notably, the study by Adachi et al.^3^ was conducted exclusively among HIV-infected pregnant women, illustrating that viral and bacterial reproductive tract infections frequently coexist in this population. Three studies focused on non-sexually transmitted infections, such as mycoplasmosis, ureaplasmosis, and bacterial vaginosis.^12,14,18^ The other three studies explored both sexually transmitted and non-sexually transmitted mono- and co-infections.^15,17,19^

The main study characteristics and abstracted data on pregnancy characteristics, maternal and neonatal outcomes, and the study conclusions are summarized in Table 1.

**Table 1.** Summary of study characteristics Table 1 long description.

Author, year (location) [considered period], Study design	Groups of interest (number of pregnancies)	Pregnancy characteristics		How were the bacterial infections diagnosed?	Did participants receive treatment for the bacterial infections during gestation?	Maternal outcomes		Neonatal outcomes		Study conclusion	Downs and black scale
		Mono-infection group(s)	Co-infection group(s)			Mono-infection group(s)	Co-infection group(s)	Mono-infection group(s)	Co-infection group(s)		
**Adachi, 2016 (Brazil, South Africa, Argentina, and the United States) [NR], Retrospective cohort (sub-study of a RCT)**	Chlamydia only (214) Gonorrhea only (28) Chlamydia and Gonorrhea (35)	**Chlamydia only**: HIV-positive women: 214 (100%) <32 wk of GA: 0 (0%) <37 wk of GA: 23 (10.8%) **Gonorrhea only**: HIV-positive women: 28 (100%) <32 wk of GA: 0 (0%) <37 wk of GA: 3 (10.7%)	**Chlamydia and Gonorrhea**: HIV-positive women: 35 (100%) <32 wk of GA: 0 (0%) <37 wk of GA: 10 (28.6%)	Gene Xpert® assay for detection of *Chlamydia trachomatis* and *Neisseria gonorrhoeae* in urine samples	No	NR		**Chlamydia only**: Any of the following: 84 (39.3%) Sepsis: 8 (3.7%) Pneumonia: 10 (4.7%) Congenital syphilis: 11 (5.1%) Septic arthritis: 0 (0%) Conjunctivitis: 2 (0.9%) Neonatal death: 4 (1.9%) Birthweight <2500g: 38 (17.8%) **Gonorrhea only**: Any of the following: 16 (57.1%) Sepsis: 3 (10.7%) Pneumonia: 4 (14.3%) Congenital syphilis: 2 (7.1%) Septic arthritis: 0 (0%) Conjunctivitis: 0 (0%) Neonatal death: 0 (0%) Birthweight <2500g: 6 (21.4%)	**Chlamydia and Gonorrhea**: Any of the following: 23 (65.7%) Sepsis: 2 (5.7%) Pneumonia: 2 (5.7%) Congenital syphilis: 3 (8.6%) Septic arthritis: 0 (0%) Conjunctivitis: 0 (0%) Neonatal death: 4 (11.4%) Birthweight <2500g: 15 (42.9%)	The combination of untreated chlamydia and/or gonorrhea infection in pregnancy and HIV exposure appears to increase the likelihood of adverse outcomes in infants beyond the risk of HIV acquisition.	19
**Ahmadi, 2024 (Iran) [2018–2023], Case–control**	Mono-infection by Mycoplasma hominis, Ureaplasma parvum, Ureaplasma urealeticum, Gardnerella vaginallis, or Streptococcus agalactiae (48) Co-infection by two of the mentioned organisms (12) Co-infection by three of the mentioned organisms (1)	Demographic characteristics were not reported specifically for the groups of interest in the current study.		PCR reaction with cervical swab samples	No	**Mono-infection**: Preterm delivery (<37 wk): 23 (47.9%)	**Co-infection by two types microrganisms**: Preterm delivery (<37 wk): 3 (25.0%) **Co-infection by three types microrganisms**: Preterm delivery (<37 wk): 0 (0%)	NR		Except for Gardnerella vaginalis bacteria, there is no significant relationship between the studied bacterial infections and spontaneous preterm birth.	15
**Felske, 2022 (the United States) [2019], Cross-sectional**	Chlamydia only (63,391) Gonorrhea only (5,759) Chlamydia and Gonorrhea (5,605) Syphilis only (4,473) Syphilis co-infection (1,181)	**Chlamydia only**: HIV-positive women: NR Induction of labor: 21,616 (34.1%) Cesarean delivery: 16,101 (25.4%) Nulliparous: 29,984 (47.3%) Pre-pregnancy hypertension: 1,458 (2.3%) Live births: 63,391 (100%) **Gonorrhea only**: HIV-positive women: NR Induction of labor: 1,808 (31.4%) Cesarean delivery: 1,756 (30.5%) Nulliparous: 2,021 (35.1%) Pre-pregnancy hypertension: 213 (3.7%) Live births: 5,759 (100%) **Syphilis only**: HIV-positive women: NR Induction of labor: 1,261 (28.2%) Cesarean delivery: 1,722 (38.5%) Nulliparous: 1,279 (28.6%) Pre-pregnancy hypertension: 219 (4.9%) Live births: 4,473 (100%)	**Chlamydia and Gonorrhea**: HIV-positive women: NR Induction of labor: 1,838 (32.8%) Cesarean delivery: 1,513 (27.0%) Nulliparous: 2,248 (40.1%) Pre-pregnancy hypertension: 157 (2.8%) Live births: 5,605 (100%) **Syphilis co-infection**: HIV-positive women: NR Induction of labor: 322 (27.3%) Cesarean delivery: 392 (33.2%) Nulliparous: 391 (33.1%) Pre-pregnancy hypertension: 51 (4.3%) Live births: 1,181 (100%)	NR	NR	**Chlamydia only**: Preterm delivery (<37 wk): 9,509 (15.0%) Gestational hypertension: 6,149 (9.7%) Eclampsia: 190 (0.3%) **Gonorrhea only**: Preterm delivery (<37 wk): 1,008 (17.5%) Gestational hypertension: 564 (9.8%) Eclampsia: 17 (0.3%) **Syphilis only**: Preterm delivery (<37 wk): 886 (19.8%) Gestational hypertension: 497 (11.1%) Eclampsia: 13 (0.3%)	**Chlamydia and Gonorrhea**: Preterm delivery (<37 wk): 1,003 (17.9%) Gestational hypertension: 493 (8.8%) Eclampsia: 17 (0.3%) **Syphilis co-infection**: Preterm delivery (<37 wk): 231 (19.6%) Gestational hypertension: 110 (9.3%) Eclampsia: 2 (0.2%)	**Chlamydia only**: Birthweight <2500g: 6,719 (10.6%) Small for GA: 4,057 (6.4%) Apgar score <7 at 5 min: 1,521 (2.4%) **Gonorrhea only**: Birthweight <2500g: 806 (14.0%) Small for GA: 403 (7.0%) Apgar score <7 at 5 min: 178 (3.1%) **Syphilis only**: Birthweight <2500g: 693 (15.5%) Small for GA: 304 (6.8%) Apgar score <7 at 5 min: 166 (3.7%)	**Chlamydia and Gonorrhea**: Birthweight <2500g: 757 (13.5%) Small for GA: 415 (7.4%) Apgar score <7 at 5 min: 151 (2.7%) **Syphilis co-infection**: Birthweight <2500g: 93 (7.9%) Small for GA: 93 (7.9%) Apgar score <7 at 5 min: 44 (3.7%)	STIs during pregnancy may be associated with adverse neonatal outcomes, especially preterm birth among individuals with syphilis infection.	20
**Kwak, 2013 (South Korea) [2005–2007], Retrospective cohort**	Ureaplasma urealyticum only (99) Ureaplasma urealyticum and Mycoplasma hominis (13)	**U. urealyticum only**: Nulliparous: 54 (54.5%) Preterm delivery: 73 (73.7%) PPROM: 46 (46.5%) Preterm delivery and/or PPROM: 99 (100%) GA at delivery (w): 34.3 ± 3.5	**U. urealyticum and M. hominis**: Nulliparous: 9 (69.2%) Preterm delivery: 13 (100%) PPROM: 9 (69.2%) Preterm delivery and/or PPROM: 13 (100%) GA at delivery (w): 29.8 ± 3.1	Cultures for U. urealyticum and M. hominis with vaginal wab samples	No	NR		**U. urealyticum only**: Birthweight: 2,393.9 ± 783.3 Small for GA: 10 (10.1%) Apgar score <7 at 5 min: 24 (24.2%) NICU admission: 53 (53.5%) Histologic chorioamnionitis: 29 (29.3%)	**U. urealyticum and M. hominis**: Birthweight: 1,521.5±384.5 Small for GA: 2 (15.4%) Apgar score <7 at 5 min: 7 (53.8%) NICU admission: 12 (92.3%) Histologic chorioamnionitis: 8 (61.5%)	Vaginal M. hominis tends to be detected with U. urealyticum, and patients carrying both organisms simultaneously had more severe adverse pregnancy outcomes compared to patients in preterm labor or PPROM who were only positive for U. urealyticum.	17
**Scoullar, 2024 (Papua New Guinea) [2015–2018], Prospective cohort**	*Mycoplasma genitalium* only (21), *Chlamydia trachomatis* only (25), *Neisseria gonorrhoeae* only (3), *Trichomonas vaginalis* only (36), Bacterial vaginosis only (36), *M. genitalium* and *N. gonorrhoeae* (3), *M. genitalium* and *C. trachomatis* (14), *M. genitalium* and *T. vaginalis* (8), *N. gonorrhoeae* and *C. trachomatis* (15), *N. gonorrhoeae* and *T. vaginalis* (3), *C. trachomatis* and *T. vaginalis* (22), *M. genitalium* and *C. trachomatis* and *N. gonorrhoeae* (3), Bacterial vaginosis and *M. genitalium* (16), Bacterial vaginosis and *C. trachomatis* (24), Bacterial vaginosis and *N. gonorrhoeae* (8), and Bacterial vaginosis and *T. vaginalis* (10)	**Entire cohort**: Primigravida: 100/416 (24.0%); fever during pregnancy: 113/416 (27.2%); liveborn singletons: 640/662 (96.7%)		With self-collected vaginal samples, the ResistancePlus was used for *M. genitalium*; the GeneXpert was used for *C. trachomatis*, *N. gonorrhoeae*, and *T. vaginalis*; Nugent scoring by microscopy was used for bacterial vaginosis	No	NR		***Mycoplasma genitalium***: Birthweight (g, mean): 2,997.0 ***Chlamydia trachomatis***: Birthweight (g, mean): 3,065.3 ***Neisseria gonorrhoeae***: Birthweight (g, mean): 2,889.2 ***Trichomonas vaginalis***: Birthweight (g): 3,146.7 ***Bacterial vaginosis***: Birthweight (g): 3,287.9	***M. genitalium* and *N. gonorrhoeae***: Birthweight (g, mean): 2,722.2 ***M. genitalium* and *C. trachomatis***: Birthweight (g, mean): 2,898.4 ***M. genitalium* and *T. vaginalis***: Birthweight (g, mean): 2,979.7 ***N. gonorrhoeae* and *C. trachomatis***: Birthweight (g, mean): 2,790.6 ***N. gonorrhoeae* and *T. vaginalis***: Birthweight (g, mean): 2,871.9 ***C. trachomatis* and *T. vaginalis***: Birthweight (g, mean): 3,048.1 ***M. genitalium* and *C. trachomatis* and *N. gonorrhoeae***: Birthweight (g, mean): 2,623.6 ***Bacterial vaginosis* and *M. genitalium***: Birthweight (g, mean): 3,121.0 ***Bacterial vaginosis* and *C. trachomatis***: Birthweight (g, mean): 3,189.3 ***Bacterial vaginosis* and *N. gonorrhoeae***: Birthweight (g, mean): 3,013.2 ***Bacterial vaginosis* and *T. vaginalis***: Birthweight (g, mean): 3,270.7	*M. genitalium* is a potential contributor to lower birthweight, and co-infections appear to have a greater negative impact on birthweight.	20
**Taylor, 2023 (the United States) [2016–2022], Retrospective cohort**	Gonorrhea only (108) Gonorrhea co-infection (107)	Demographic characteristics were not reported specifically for the groups of interest in the current study.		Vaginal swabs or urine were used for *C. trachomatis* and *N. gonorrhoeae* using nucleic acid amplification tests; syphilis was diagnosed with rapid plasma regain with blood samples	Presumably yes	**Gonorrhea only**: Hypertensive pregnancy disorders: 19 (17.6%) Superimposed preeclampsia: 2 (1.9%) Preeclampsia: 9 (8.3%) Gestational hypertension: 11 (10.2%) Preterm preeclampsia: 5 (4.6%) Term preeclampsia: 4 (3.7%) Preeclamsia with severe features: 6 (5.6%) Preterm delivery (<37 wk): 17 (15.7%) PPROM: 2 (1.9%)	**Gonorrhea co-infection**: Hypertensive pregnancy disorders: 13 (12.2%) Superimposed preeclampsia: 0 (0%) Preeclampsia: 9 (8.4%) Gestational hypertension: 5 (4.7%) Preterm preeclampsia: 5 (4.7%) Term preeclampsia: 4 (3.7%) Preeclamsia with severe features: 3 (2.8%) Preterm delivery (<37 wk): 16 (15.9%) PPROM: 4 (3.7%)	**Gonorrhea only**: Chorioamnionitis: 3 (2.8%)	**Gonorrhea co-infection**: Chorioamnionitis: 3 (2.8%)	Among a diverse population of pregnant individuals, gonorrhea increased odds of preterm preeclampsia and preterm delivery.	16
**Yao, 2022 (the United States) [2007–2012], Retrospective cohort**	Chlamydia only (6,822), Gonorrhea only (8,932), Chlamydia and Gonorrhea (184), Bacterial vaginosis (2,113)	**Entire cohort**: Singleton pregnancies: 18,051 (100%)		NR	NR	**Chlamydia only**: Preterm delivery (<37 wk): 905 (13.3%) Preterm delivery (<34 wk): 476 (7.0%) PPROM: 366 (5.4%) Abruption: 93 (1.4%) **Gonorrhea only**: Preterm delivery (<37 wk): 1,352 (15.1%) Preterm delivery (<34 wk): 628 (7.0%) PPROM: 479 (5.4%) Abruption: 133 (1.5%) **Bacterial vaginosis**: Preterm delivery (<37 wk): 605 (28.6%) Preterm delivery (<34 wk): 427 (20.2%) PPROM: 182 (8.6%) Abruption: 90 (4.3%)	**Chlamydia and Gonorrhea**: Preterm delivery (<37 wk): 32 (17.4%) Preterm delivery (<34 wk): 20 (10.9%) PPROM: 11 (6.0%) Abruption: 4 (2.2%)	**Chlamydia only**: Intra-amniotic infection: 205 (3.0%) **Gonorrhea only**: Intra-amniotic infection: 240 (2.7%) **Bacterial vaginosis**: Intra-amniotic infection: 178 (8.4%)	**Chlamydia and Gonorrhea**: Intra-amniotic infection: 4 (2.2%)	Prenatal bacterial vaginosis infection was associated with higher risks of preterm complications including preterm delivery, PPROM, Triple I and abruption compared with gonorrhea and chlamydia infection.	10
**Yoneda, 2016 (Japan) [2001–2014], Retrospective cohort**	Mycoplasma or Ureaplasma only (7) Mycoplasma or Ureaplasma and other bacteria (17)	**Mycoplasma or Ureaplasma only**: Nulliparity: 4 (57.1%) Gestational age at amniocentesis (w): 26 (22–27) Antenatal corticosteroid: 3 (42.9%) Preterm labor: 7 (100%) Singleton pregnancies: 7 (100%)	**Mycoplasma or Ureaplasma and other bacteria**: Nulliparity: 7 (41.2%) Gestational age at amniocentesis (w): 24 (18–33) Antenatal corticosteroid: 7 (41.2%) Preterm labor: 17 (100%) Singleton pregnancies: 17 (100%)	PCR reaction and culture with amniotic fluid sample; levels of IL-8 in the amniotic fluid measured by enzyme-linked immunosorbent assay (ELISA); placental histological examination; metagenomic sequencing of 16S rRNA gene; operational taxonomic units (OTUs) analysis of metagenomic sequencing	No	**Mycoplasma or Ureaplasma only**: GA at delivery: 28 (23–37)	**Mycoplasma or Ureaplasma and other bacteria**: GA at delivery: 25 (22–37)	**Mycoplasma or Ureaplasma only**: Birthweight: 1,540 ± 631 Apgar score <7 at 1 min: 2 (28.6%) Apgar score <7 at 5 min: 1 (14.3%) Umbilicar artery pH at birth: 7.286 ± 0.08 Stillbirth: 0 (0%) Deaths at perinatal period (<28 d): 0 (0%) NICU admission: 6 (85.7%) Composite severe neonatal morbidity: 1 (14.3%) Congenital neonatal sepsis: 0 (0%) Respiratory distress syndrome: 4 (57.1%) Necrotizing enterocolitis: 0 (0%) Intraventricular hemorrhage: 1 (14.3%) Bronchopulmonary dysplasia: 0 (0%)	**Mycoplasma or Ureaplasma and other bacteria**: Birthweight: 1,219 ± 643 Apgar score <7 at 1 min: 10/15 (66.6%) Apgar score <7 at 5 min: 6/15 (40.0%) Umbilicar artery pH at birth: 7.312 ± 0.06 Stillbirth: 2/17 (0%) Deaths at perinatal period (<28 d): 1/15 (6.7%) NICU admission: 14/15 (93.3%) Composite severe neonatal morbidity: 6/15 (40.0%) Congenital neonatal sepsis: 0/15 (0%) Respiratory distress syndrome: 7/15 (46.7%) Necrotizing enterocolitis: 1/15 (6.7%) Intraventricular hemorrhage: 2/15 (13.3%) Bronchopulmonary dysplasia: 5/15 (33.3%)	Polymicrobial infection with Mycoplasma/Ureaplasma and other bacteria induces severe intra-amniotic inflammation associated with poor perinatal prognosis in preterm labor.	17
**Zeng, 2022 (China) [2010–2016], Retrospective cohort**	Trichomoniasis independent (142) Trichomoniasis co-infection (42) Syphilis independent (306) Syphilis co-infection (42) Bacterial vaginosis independent (367) Bacterial vaginosis co-infection (40) Chlamydia independent (99) Chlamydia co-infection (26)	Demographic characteristics were not reported specifically for the groups of interest in the current study.		Golden colloid immunity diffuse test for the diagnosis of *C. trachomatis*; trichomoniasis was diagnosed by microscopic identification of typical motile; syphilis was diagnosed with rapid plasma regain; bacterial vaginosis was identified by the Amsel criteria.	Presumably yes	**Trichomoniasis independent**: Spontaneous abortion: 11/142 (7.8%) **Syphilis independent**: Spontaneous abortion: 38/306 (12.4%) Induced abortion/labor due to medical reasons: 20/306 (6.5%) Preterm delivery (<37 wk): 30/242 (12.4%) **Bacterial vaginosis independent**: Very preterm delivery (<32 wk): 10/367 (2.7%)	**Trichomoniasis co-infection**: Spontaneous abortion: 7/42 (16.7%) **Syphilis co-infection**: Spontaneous abortion: 7/42 (16.7%) Induced abortion/labor due to medical reasons: 0/42 (0%) Preterm delivery (<37 wk): 4/34 (11.8%) **Bacterial vaginosis co-infection**: Very preterm delivery (<32 wk): 3/40 (7.5%)	**Bacterial vaginosis independent**: Macrosomia: 24/365 (6.6%) Large for GA: 49/365 (13.4%) **Chlamydia independent**: Macrosomia: 11/99 (11.1%)	**Bacterial vaginosis co-infection**: Macrosomia: 3/40 (7.5%) Large for GA: 6/40 (15.0%) **Chlamydia co-infection**: Macrosomia: 0/26 (0%)	Preconception reproductive tract infectins, such as syphilis and bacterial vaginosis, were associated with increased risks of many adverse pregnancy outcomes including spontaneous abortion, preterm birth and macrosomia	18

NR, not reported; RCT, randomized clinical trial; GA, gestational age; HIV, human immunodeficiency virus; STI, sexually transmitted infection; PPROM, preterm premature rupture of membranes; NICU, neonatal intensive care unit; PCR, polymerase chain reaction; RNA, ribonucleic acid.

### Quality assessment

Regarding the quality assessment, four studies were considered of high quality per the Downs and Black quality tool,^3,13,15,19^ while four other were deemed fair quality,^12,14,16,18^ and one, which was an abstract, poor quality^17^ (Table 1).

The funnel plot appeared symmetrical, and Egger’s test did not indicate evidence of publication bias among the five studies included in the prenatal delivery analysis^12,13,16,17,19^ (*P* value .07, Supplemental Figure 1).

### Maternal outcomes

**Figure 2.**
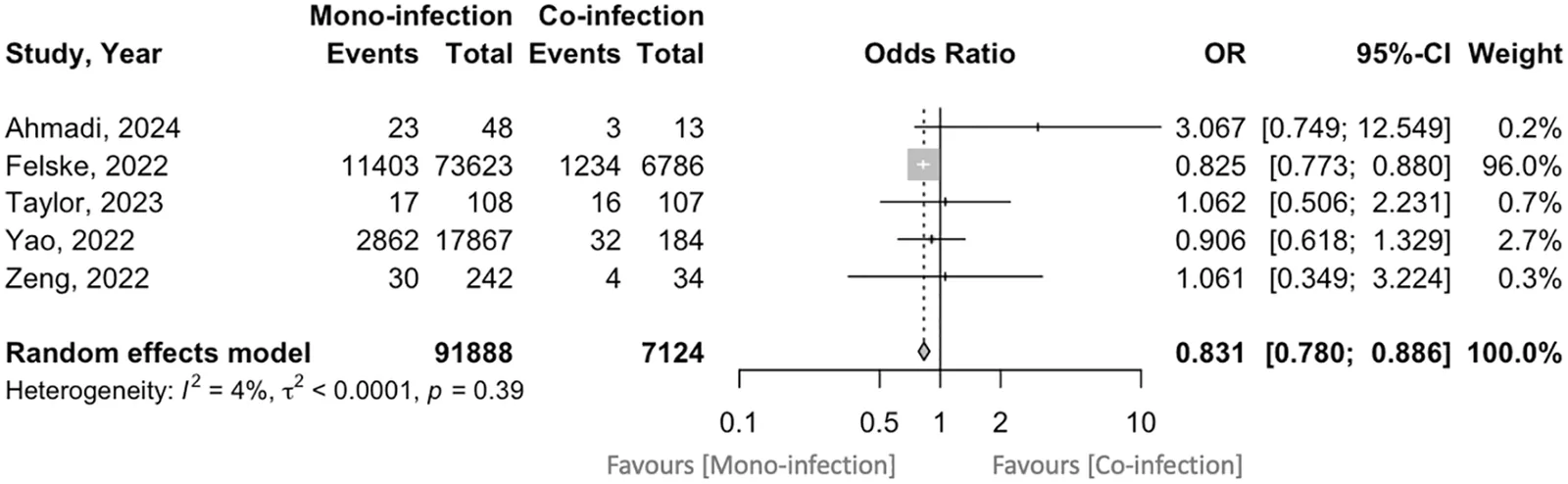
Forest plot of preterm delivery (<37 wk of gestational age) events according to the number of maternal non-viral reproductive tract infections. ORs were determined with the Mantel–Haenszel random-effects method. OR, odds ratio; CI, confidence interval.

Five studies reported prenatal delivery, defined as birth before the completion of the 37^th^ week of gestation, by infection status.^12,13,16,17,19^ The analysis of these five showed statistically significant advantage toward any mono-infection group (pooled OR 0.83, 95% CI 0.78–0.89, *I* ^2^ = 4%, Figure 2) compared to any co-infection group. Excluding the large cohort study by Felske et al.^13^ which alone surpassed the weight of 95%, there was no statistical difference between groups (pooled OR 1.01, 95% CI 0.73–1.38, *I* ^2^ = 0%, Supplemental Figure 2) among the remaining four studies.^12,16,17,19^ Two studies reported birth before the completion of the 34^th^ week of gestation as an outcome.^17,19^ No statistically significant difference was found between the two groups (pooled OR 0.66, 95% CI 0.36–1.22, *I* ^2^ = 19%, Supplemental Figure 3).

Two studies compared chlamydia mono-infection and chlamydia co-infection in preterm delivery (<37 wk).^13,17^ In these two studies, the co-infection group was formed by patients co-infected with chlamydia and gonorrhea. In this analysis, there was a statistically significant advantage toward the mono-infection group (pooled OR 0.81, 95% CI 0.75–0.87, *I* ^2^ = 0%, Supplemental Figure 4).^13,17^ Three studies compared the rate of preterm delivery (<37 wk) of gonorrhea mono-infection and gonorrhea co-infection groups.^13,16,17^ The analysis showed no difference (pooled OR 0.97, 95% CI 0.88–1.06, *I* ^2^ = 0%, Supplemental Figure 5). When analyzing preterm delivery (<37 wk) in syphilis mono-infection and co-infection groups, no statistical difference was observed (pooled OR 1.02, 95% CI 0.87–1.19, *I* ^2^ = 0%, Supplemental Figure 6).

Two studies evaluated the rate of gestational hypertension according to infection status.^13,16^ Meta-analysis showed no significant difference between mono-infection and co-infection (pooled OR 1.30, 95% CI 0.72–2.33, *I* ^2^ = 42%, Supplemental Figure 7). Two studies compared both gestational hypertension and eclampsia or preeclampsia with severe features specifically in gonorrhea mono-infection and gonorrhea co-infection groups, with no statistical difference found (pooled OR 1.30, 95% CI 0.74–2.30, *I* ^2^ = 39% and pooled OR 1.12, 95% CI 0.61–2.05, *I* ^2^ = 0%, respectively, Supplemental Figures 9 and 10).^13,16^

Two studies evaluated the rate of PPROM according to the number of reproductive tract infections.^16,17^ Meta-analysis showed no significant difference between mono-infections and co-infections (pooled OR 0.89, 95% CI 0.50–1.58, *I* ^2^ = 0%, Supplemental Figure 11). An analysis limited to the comparison of PPROM rates in gonorrhea mono-infection and co-infection showed no difference also (pooled OR 0.83, 95% CI 0.47–1.49, *I* ^2^ = 0%, Supplemental Figure 12).

### Neonatal outcomes

Spontaneous abortion or stillbirth rates were reported by two studies.^18,19^ Meta-analysis showed no statistically significant difference between the two groups (pooled OR 0.60, 95% CI 0.32–1.14, *I* ^2^ = 0%, Supplemental Figure 13). Neonatal death was also reported by two studies, with statistically significant advantage toward the mono-infections group (pooled OR 0.17, 95% CI 0.05–0.63, *I* ^2^ = 0%, Supplemental Figure 14) compared to any co-infection group.

**Figure 3.**
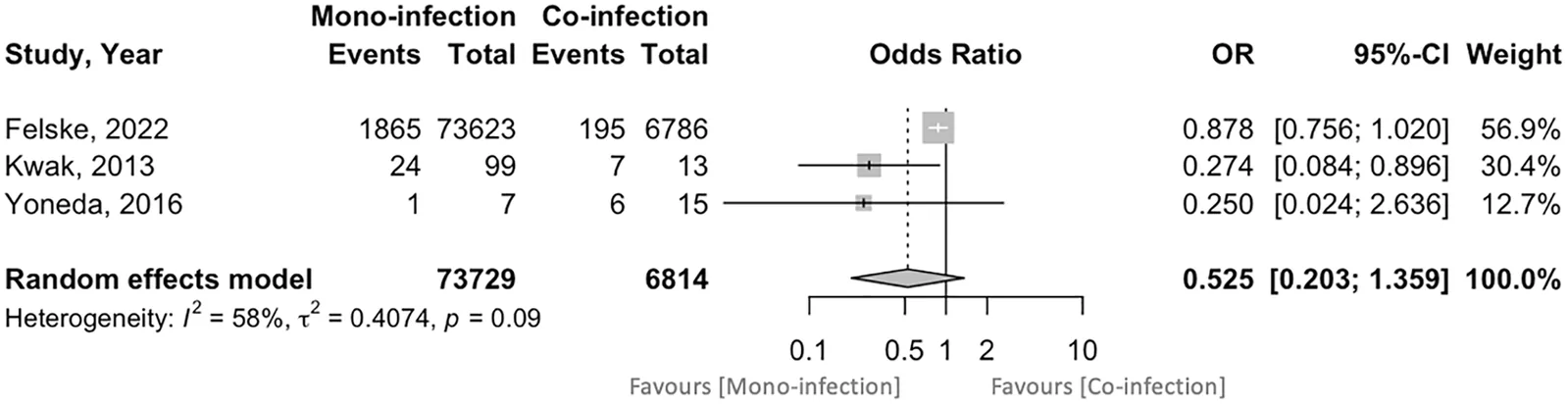
Figure 3 long description. Forest plot of Apgar score <7 at five minutes events according to the number of maternal reproductive tract non-viral infections. ORs were determined with the Mantel–Haenszel random-effects method. OR, odds ratio; CI, confidence interval.

Two studies evaluated the need for neonatal intensive care unit admission.^14,18^ Meta-analysis revealed a significant advantage for the mono-infection group of patients (pooled OR 0.16, 95% CI 0.03–0.86, *I* ^2^ = 0%, Supplemental Figure 15). Apgar score <7 at five minutes was evaluated by three studies.^13,14,18^ Meta-analysis showed no difference between groups (pooled OR 0.53, 95% CI 0.20–1.36, *I* ^2^ = 58%, Figure 3).

**Figure 4.**
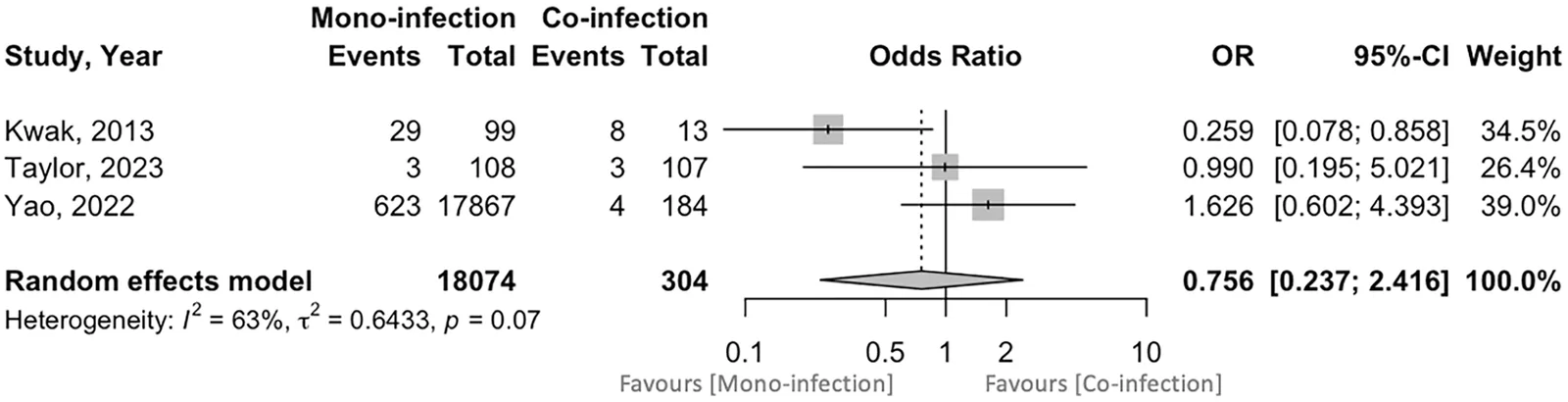
Forest plot of chorioamnionitis or intra-amniotic infection events according to the number of maternal reproductive tract non-viral infections. ORs were determined with the Mantel–Haenszel random-effects method. OR, odds ratio; CI, confidence interval.

Chorioamnionitis or intra-amniotic infection was reported by three studies.^14,16,17^ No statistical difference was noted between the mono-infection and co-infection groups (pooled OR 0.76, 95% CI 0.24–2.42, *I* ^2^ = 63%, Figure 4). Comparing only gonorrhea mono-infection with gonorrhea co-infection, no difference was also found (pooled OR 1.17, 95% CI 0.50–2.73, *I* ^2^ = 0%, Supplemental Figure 16).^16,17^ Two studies reported neonatal sepsis rates by number of bacterial infections.^3,18^ One study without cases of neonatal sepsis could not be included in the random-effects model.^18^ The analysis of the one remaining study showed no significant difference in neonatal sepsis rates between groups (pooled OR 0.79, 95% CI 0.17–3.70, Supplemental Figure 17).

Five studies reported data on birthweight.^3,13–15,18^ Two of these studies presented the data qualitatively, indicating the number of neonates with a birthweight <2,500 g.^3,13^ A meta-analysis of these two studies revealed no statistically significant difference between the mono-infection and co-infection groups (pooled OR 0.55, 95% CI 0.19–1.57, *I* ^2^ = 88%, Supplemental Figure 18). The other three studies reported the mean birthweight of neonates in grams.^14,15,18^ Of these, two provided standard deviations,^14,18^ allowing them to be included in a meta-analysis, which showed an advantage for the mono-infection group (total mean difference 625.78, 95% CI 89.41–1,162.14, *I* ^2^ = 57%, Supplemental Figure 19). Coincidentally, these two studies evaluated non-sexually transmitted infections caused by the same organisms, *Ureaplasma* and *Mycoplasma*.

Comparing the rates of birthweight <2,500 g in chlamydia mono-infection and chlamydia co-infection groups, the meta-analysis of two studies showed no statistical difference (pooled OR 0.50, 95% CI 0.20–1.29, *I* ^2^ = 84%, Supplemental Figure 20).^3,13^ Both studies considered chlamydia and gonorrhea co-infection in the co-infection group. An analysis limited to the comparison of birthweight <2,500 in gonorrhea mono-infection and co-infection showed no difference also (pooled OR 0.72, 95% CI 0.27–1.93, *I* ^2^ = 70%, Supplemental Figure 21).^3,13^

Two studies evaluated the rate of newborns small for GA at birth.^13,14^ Meta-analysis showed a slight, but significant, statistical advantage for the mono-infection group (pooled OR 0.85, 95% CI 0.78–0.94, *I* ^2^ = 0%, Supplemental Figure 22). Even though the heterogeneity was acceptable, the study with more participants weighted more than 99%.

The outcomes of analyses comparing any co-infection with any mono-infection are summarized in Supplemental Table 1, while the comparisons between co-infections and specific mono-infections are presented in Supplemental Table 2.

## Discussion

### Main findings

In this systematic literature review and meta-analysis, reproductive tract non-viral mono-infections were associated with lower rates of adverse maternal and neonatal outcomes, including preterm delivery, neonatal intensive care unit admission, and neonatal death, compared to non-viral co-infections. However, the analysis did not reveal significant differences between the groups in terms of gestational hypertension, eclampsia, spontaneous abortion or stillbirth, and chorioamnionitis.

This study represents the most extensive systematic review and meta-analysis conducted to date of reproductive tract non-viral mono-infections and co-infections during pregnancy. Our findings indicate that co-infections may exacerbate certain neonatal outcomes while having a similar impact on maternal complications compared to mono-infections. However, the studies did not assess the potential long-term effects of bacterial infections on maternal health beyond the gestation period.

Despite the high incidence of reproductive tract bacterial infections in pregnant women and the well-documented risks they pose to newborns,^20–23^ there is a notable lack of studies comparing outcomes between mono-infections and co-infections during gestation. Our analyses revealed statistically significant differences between the two groups only in the rates of prenatal delivery, neonatal death, neonatal intensive care unit admission, and low birthweight, all of which favored mono-infections over co-infections. Among analysis of specific infections, only preterm birth showed a statistically significant difference, favoring the chlamydia mono-infection group over the chlamydia co-infection group.

### Strengths and limitations

To ensure comprehensiveness, we included studies regardless of the GA at birth, HIV serologic status, or the number of fetuses. Whenever mentioned by the study, we described these baseline characteristics. Stratified analyses could not be performed based on publication date, location, study design, diagnosis, treatment, birthweight, and GA, because of the number of studies. Even when analyzing all studies that reported each of the outcomes, considering all infections, the number of studies for each analysis was low (only two or three). Conducting stratified analyses based on demographic characteristics would have resulted in a negligible sample size, with only one study per analysis. To enable some of the analyses, we combined similar outcomes reported by different studies into composite outcomes, such as eclampsia and preeclampsia with severe features, or spontaneous abortion and stillbirth.

Our study has several limitations. First, the included studies were non-randomized, which inherently increases the risk of both measured and unmeasured confounding factors, such as maternal age, socioeconomic status, and underlying comorbidities. However, given the ethical challenges of conducting randomized prospective studies to assess the risks of co-infection and mono-infection, non-randomized retrospective designs were likely the most appropriate and feasible approach. Furthermore, adjusted ORs controlling for factors such as GA and HIV serological status were not possible due to the lack of individual participant data and inconsistent reporting across studies. This limitation is crucial because the GA at infection significantly affects outcomes. For instance, during the early stages of pregnancy, bacterial infections and antibiotic treatment can markedly increase the risk of miscarriage and birth defects; in the third trimester, preterm labor and premature rupture of membranes become the primary concerns.^24,25^ Second, not all studies examined all outcomes, leading to low numbers available for the analyses. Consequently, robust statistical assessments for less-studied events like birthweight <2,500 g, chorioamnionitis, and neonatal sepsis were hindered. Thirdly, analyses of rare but clinically important outcomes may lack statistical power, as seen in reductions in spontaneous abortion or stillbirth (65% reduction) and delivery before the 34^th^ week of gestation (50% reduction) in the mono-infection group, which were not statistically significant. Nonsignificant results do not necessarily indicate the absence of an association but may reflect limited sample size, low statistical power, or the presence of confounding variables. Fourth, due to the limited number of studies available for several comparisons, we were unable to perform further stratified analyses by study design or geographic region, as such subgrouping would have compromised the statistical power and interpretability of the findings. Fifth, heterogeneity among studies may undermine the generalizability of conclusions, particularly in less-studied scenarios. Sixth, diagnostic criteria varied across primary studies. For *Ureaplasma spp*. and *Mycoplasma hominis*, diagnosis relied on PCR or culture positivity rather than validated quantitative thresholds; since these organisms are common commensals, PCR detection does not confirm clinically significant infection, and no study reported bacterial load or cycle-threshold values to distinguish true infection from colonization. This diagnostic ambiguity, inherent to the primary literature, may have caused non-differential misclassification affecting our pooled estimates for these organisms.

The findings are particularly relevant for obstetricians during antenatal counseling. Since women with one bacterial reproductive tract infection commonly have others,^26^ early diagnosis and investigation of additional infections is essential. If a single infection can harm both the mother and newborn, co-infections are associated with higher rates of adverse outcomes, though further research is needed to confirm causality.

### Correlation with existing literature

Gonorrhea infection has been associated with preterm birth, premature rupture of membranes, perinatal mortality, and low birthweight^4^ and *Chlamydia* mono-infection with fetal death and preterm birth.^27^ Given the frequency of bacterial co-infections,^3^ systematically review which pregnancy outcomes are most affected by the number of non-viral infections was essential. To our knowledge, this is the most comprehensive systematic review and meta-analysis comparing non-viral mono-infections and co-infections during pregnancy, and the only one considering both maternal and neonatal outcomes. Previous studies have examined the impact of co-infections, in general, during gestation. It has been reported that pregnancies complicated by HIV have a higher risk of adverse outcomes, such as stillbirth and HIV vertical transmission, when co-infected with syphilis or tuberculosis.^28–30^ There are also indications that ToRCH (toxoplasmosis, rubella, cytomegalovirus, and herpes simplex virus) co-infections may be associated with a higher risk of spontaneous abortion compared to single infections.^31^

Beyond non-viral co-infections, interactions between viral and bacterial reproductive tract pathogens are well documented. The “epidemiological synergy” between HIV and bacterial STIs such as chlamydia, gonorrhea, and syphilis is well established: bacterial-induced genital inflammation increases both HIV shedding and acquisition risk, and treating the bacterial infection reduces this risk.^32^ Notably, one of our nine included studies was conducted exclusively in HIV-infected pregnant women evaluating chlamydia/gonorrhea mono- and co-infection.^3^ Together with the HIV-syphilis, HIV-tuberculosis, and ToRCH data discussed above,^28–31^ these findings support the biological plausibility that co-infections, whether non-viral or viral-bacterial, may compound adverse outcomes through shared mechanisms of mucosal inflammation and impaired local immunity.

Congenital syphilis is a well-established risk factor for fetal and neonatal death, preterm birth, low birthweight, and extended hospital stays.^33,34^ Given the rising number of syphilis cases among women of reproductive age,^35–37^ our review underscores the need for further studies that examine co-infections of syphilis with other bacterial infections.

The findings of this study, along with previous research, reinforce the importance of screening for other infectious diseases once one is diagnosed during pregnancy, whether bacterial, viral, or parasitic.

Recent research^38^ has demonstrated that *Chlamydia* may be associated with gestational hypertension, preeclampsia with severe features, and superimposed preeclampsia. In contrast, gonorrhea was not found to be associated with hypertensive disorders of pregnancy. Nevertheless, although gonorrhea may not directly elevate the risk of hypertensive disorders, it could still contribute to the development of these conditions in certain cases. More studies are needed to clarify this relationship.

In the latest Sexually Transmitted Infections Treatment Guidelines,^39^ the Centers for Disease Control and Prevention (CDC) emphasize the importance of screening for bacterial sexually transmitted infections in pregnant women. Syphilis screening is recommended at the first prenatal visit, with additional testing in the third trimester and at delivery for women at high risk. Timely detection and treatment are essential to prevent congenital syphilis, which has significantly increased in the United States. Similarly, screening for chlamydia and gonorrhea is advised for all pregnant women under 25 years old and those at increased risk. Women with ongoing risk factors should be retested later in pregnancy to avoid maternal and neonatal complications. Immediate treatment of positive cases is crucial, followed by a test of cure to ensure eradication. Although evidence does not support routine screening for asymptomatic bacterial vaginosis in pregnant women, those showing symptoms should be evaluated and treated accordingly. The focus is on individualized care, early detection, prompt treatment to minimize adverse outcomes such as preterm delivery and neonatal infections.

## Conclusion

Compared to reproductive tract non-viral mono-infections, co-infections appear to be associated with higher rates of adverse maternal and neonatal outcomes, particularly an increased risk of preterm delivery, neonatal intensive care unit admission, and neonatal death. However, other outcomes, such as chorioamnionitis, PPROM, and preterm birth before 34 weeks, did not show significant differences between the groups. Given the variability of findings and the potential influence of confounding factors, future studies with larger sample sizes and multivariate adjustment models are needed to establish a more precise correlation between the number of infections and pregnancy outcomes.

## Supporting information

10.1017/ash.2026.10821.sm001 Callado et al. supplementary material Callado et al. supplementary material

## Supplementary material

The supplementary material for this article can be found at https://doi.org/10.1017/ash.2026.10821.

## Figure 1. Long description

A flowchart illustrating the process of selecting articles for a meta-analysis on the impact of non-viral reproductive tract infections on maternal and neonatal outcomes. The flowchart begins with the identification phase, where a total of 1,436 articles are identified from various databases: MEDLINE (303), CINAHL (69), Embase (394), Cochrane (52), Scopus (448), and Web of Science (170). Duplicates are removed, resulting in 457 duplicates being excluded. This leaves 979 records to be screened. During the screening phase, 945 articles are excluded based on the examination of titles or abstracts. This results in 34 full-text articles being assessed for eligibility. In the eligibility phase, 25 articles are excluded for various reasons: 2 are reviews, 13 did not report outcomes, 3 did not study co-infections, 3 studied only mono-infections, 4 studied only co-infections, 2 took viral infections into consideration in the co-infection groups, and 1 was not about pregnancy. This leaves 9 studies included in the qualitative synthesis. Finally, 8 articles are included in the quantitative synthesis (meta-analysis) evaluating maternal or neonatal outcomes.

Navigate back to Figure 1..

## Table 1. Long description

A table with 10 rows and 10 columns summarizing study characteristics and data on pregnancy outcomes. The columns include Study, Design, Population, Sample size, Exposure, Outcome, Results, Conclusions, and Notes. Each row provides detailed information for different studies. For example, Row 1 includes: Study: Smith et al., Design: Cohort study, Population: Pregnant women, Sample size: 500, Exposure: Gestational diabetes, Outcome: Neonatal hypoglycemia, Results: Increased risk, Conclusions: Gestational diabetes increases neonatal hypoglycemia risk, Notes: DOI: 10.1001/jama.2020.1234.

Navigate back to Table 1..

## Figure 3. Long description

A table comparing odds ratios of Apgar score events between mono-infection and co-infection across different studies. The table has 4 rows and 8 columns. The columns are labeled Study, Year, Mono-infection Events, Mono-infection Total, Co-infection Events, Co-infection Total, Odds Ratio, OR, 95%-CI, and Weight. The rows are labeled with different studies and their respective data. Row 1: Felske, 2022, 1865, 73623, 195, 6786, 0.878, 0.756, 1.020, 56.9. Row 2: Kwak, 2013, 24, 99, 7, 13, 0.274, 0.084, 0.896, 30.4. Row 3: Yoneda, 2016, 1, 7, 6, 15, 0.250, 0.024, 2.636, 12.7. Row 4: Random effects model, 73729, 6814, 0.525, 0.203, 1.359, 100.0. The table also includes a forest plot showing the odds ratios and their confidence intervals.

Navigate back to Figure 3..
