# Supplementary material for: Reproductive tract non-viral mono- and co-infections in pregnancy: systematic literature review and meta-analysis
Source: Antimicrob Steward Healthc Epidemiol. 2026 Sep 8;6(1):e260. doi: 10.1017/ash.2026.10821 (PMC13760399; doi:10.1017/ash.2026.10821)
Supplement: Supplementary file 1 [file S2732494X26108213sup001.docx]

**Reproductive Tract Non-Viral Mono- and Co-Infections in Pregnancy: Systematic Literature Review and Meta-Analysis**

Supplemental Material

**Supplemental Search Strategy:**

(((((((("pregnant women"[MeSH Terms]) OR ("pregnancy"[MeSH Terms])) OR ("pregnancy complications"[MeSH Terms])) OR ("pregnancy complications, infectious"[MeSH Terms])) AND (((((((((((((((((((((((("sexually transmitted diseases"[MeSH Terms]) OR ("sexually transmitted diseases, bacterial"[MeSH Terms])) OR ("chancroid"[MeSH Terms])) OR ("chlamydia infections"[MeSH Terms])) OR ("gonorrhea"[MeSH Terms])) OR ("granuloma inguinale"[MeSH Terms])) OR ("syphilis"[MeSH Terms])) OR ("sexually transmitted diseases, viral"[MeSH Terms])) OR ("condylomata acuminata"[MeSH Terms])) OR ("herpes genitalis"[MeSH Terms])) OR ("hiv infections"[MeSH Terms])) OR ("papillomavirus infections"[MeSH Terms])) OR ("mycoplasma"[MeSH Terms])) OR ("chlamydia"[MeSH Terms])) OR ("reproductive tract infections"[MeSH Terms])) OR ("trichomonas infections"[MeSH Terms])) OR ("chlamydia trachomatis"[MeSH Terms])) OR ("neisseria gonorrhoeae"[MeSH Terms])) OR ("mycoplasma genitalium"[MeSH Terms])) OR ("trichomonas vaginalis"[MeSH Terms])) OR ("trichomonas vaginitis"[MeSH Terms])) OR ("mycoplasma hominis"[MeSH Terms])) OR ("ureaplasma urealyticum"[MeSH Terms])) OR ("ureaplasma"[MeSH Terms]))) AND ("coinfection"[MeSH Terms])) AND (((("chorioamnionitis"[MeSH Terms]) OR ("birth weight"[MeSH Terms])) OR ("premature birth"[MeSH Terms])) OR ("stillbirth"[MeSH Terms]))) OR ((((((((((((((("pregnant women"[Title/Abstract]) OR ("pregnancy"[Title/Abstract])) OR ("pregnancy complications"[Title/Abstract])) OR ("infectious pregnancy complications"[Title/Abstract])) OR ("pregnant"[Title/Abstract])) OR ("gestation"[Title/Abstract])) OR ("maternal"[Title/Abstract])) OR ("conception"[Title/Abstract])) OR ("preconception"[Title/Abstract])) OR ("pre conception"[Title/Abstract])) OR ("preconceptions"[Title/Abstract])) OR ("pre conceptions"[Title/Abstract])) AND ((((((((((((((((((((((((((((((((((((((((("sexually transmitted diseases"[Title/Abstract]) OR ("bacterial sexually transmitted diseases"[Title/Abstract])) OR ("chancroid"[Title/Abstract])) OR ("chlamydia infections"[Title/Abstract])) OR ("chlamydia infection"[Title/Abstract])) OR ("gonorrhea"[Title/Abstract])) OR ("granuloma inguinale"[Title/Abstract])) OR ("syphilis"[Title/Abstract])) OR ("viral sexually transmitted diseases"[Title/Abstract] OR "viral sexually transmitted infection"[Title/Abstract])) OR ("bacterial sexually transmitted infections"[Title/Abstract])) OR ("bacterial sexually transmitted diseases"[Title/Abstract])) OR ("bacterial sexually transmitted disease"[Title/Abstract])) OR ("condylomata"[Title/Abstract])) OR ("condylomata acuminata"[Title/Abstract])) OR ("herpes genitalis"[Title/Abstract])) OR ("hiv infections"[Title/Abstract])) OR ("hiv infection"[Title/Abstract])) OR ("papillomavirus infections"[Title/Abstract])) OR ("papillomavirus infection"[Title/Abstract])) OR ("mycoplasma"[Title/Abstract])) OR ("chlamydia"[Title/Abstract])) OR ("reproductive tract infections"[Title/Abstract])) OR ("reproductive tract infection"[Title/Abstract])) OR ("trichomonas infections"[Title/Abstract])) OR ("trichomonas infection"[Title/Abstract])) OR ("chlamydia trachomatis"[Title/Abstract])) OR ("neisseria gonorrhoeae"[Title/Abstract])) OR ("mycoplasma genitalium"[Title/Abstract])) OR ("trichomonas vaginalis"[Title/Abstract])) OR ("trichomonas vaginitis"[Title/Abstract])) OR ("mycoplasma hominis"[Title/Abstract])) OR ("ureaplasma urealyticum"[Title/Abstract])) OR ("ureaplasma"[Title/Abstract])) OR ("sexually transmitted infections"[Title/Abstract])) OR ("sexually transmitted infection"[Title/Abstract])) OR ("trichomoniasis"[Title/Abstract])) OR ("vaginosis"[Title/Abstract])) OR ("ureaplasma parvum"[Title/Abstract])) OR ("n gonorrhoeae"[Title/Abstract])) OR ("viral sexually transmitted infection"[Title/Abstract] OR "viral sexually transmitted infections"[Title/Abstract])) OR (bacterial sexually transmitted disease[Title/Abstract]))) AND (((((((("coinfection"[Title/Abstract]) OR ("coinfections"[Title/Abstract])) OR ("co infection"[Title/Abstract])) OR ("co infections"[Title/Abstract])) OR ("monoinfection"[Title/Abstract])) OR ("monoinfections"[Title/Abstract])) OR ("mono infection"[Title/Abstract])) OR ("mono infections"[Title/Abstract]))) AND (((((((((((((((((((((((("chorioamnionitis"[Title/Abstract]) OR ("birth weight"[Title/Abstract])) OR ("premature birth"[Title/Abstract])) OR ("stillbirth"[Title/Abstract])) OR ("adverse outcomes"[Title/Abstract])) OR ("adverse outcome"[Title/Abstract])) OR ("adverse pregnancy outcomes"[Title/Abstract])) OR ("adverse pregnancy outcome"[Title/Abstract])) OR ("birthweight"[Title/Abstract])) OR ("macrosomia"[Title/Abstract])) OR ("maternal outcomes"[Title/Abstract])) OR ("maternal outcome"[Title/Abstract])) OR ("outcomes"[Title/Abstract])) OR ("outcome"[Title/Abstract])) OR ("lower birthweight"[Title/Abstract])) OR ("lower birth weight"[Title/Abstract])) OR ("preterm"[Title/Abstract])) OR ("adverse neonatal"[Title/Abstract])) OR ("adverse maternal outcomes"[Title/Abstract])) OR ("adverse maternal outcome"[Title/Abstract])) OR ("preterm birth"[Title/Abstract])) OR ("abortion"[Title/Abstract])) OR ("pregnancy outcome"[Title/Abstract])) OR ("pregnancy outcomes"[Title/Abstract])))) OR ((((((((((((((("pregnant women"[Text Word]) OR ("pregnancy"[Text Word])) OR ("pregnancy complications"[Text Word])) OR ("infectious pregnancy complications"[Text Word])) OR ("pregnant"[Text Word])) OR ("gestation"[Text Word])) OR ("maternal"[Text Word])) OR ("conception"[Text Word])) OR ("preconception"[Text Word])) OR ("pre conception"[Text Word])) OR ("preconceptions"[Text Word])) OR ("pre conceptions"[Text Word])) AND ((((((((((((((((((((((((((((((((((((((((("sexually transmitted diseases"[Text Word]) OR ("bacterial sexually transmitted diseases"[Text Word])) OR ("chancroid"[Text Word])) OR ("chlamydia infections"[Text Word])) OR ("chlamydia infection"[Text Word])) OR ("gonorrhea"[Text Word])) OR ("granuloma inguinale"[Text Word])) OR ("syphilis"[Text Word])) OR ("viral sexually transmitted diseases"[Text Word] OR "viral sexually transmitted infection"[Text Word])) OR ("bacterial sexually transmitted infections"[Text Word])) OR ("bacterial sexually transmitted diseases"[Text Word])) OR ("bacterial sexually transmitted disease"[Text Word])) OR ("condylomata"[Text Word])) OR ("condylomata acuminata"[Text Word])) OR ("herpes genitalis"[Text Word])) OR ("hiv infections"[Text Word])) OR ("hiv infection"[Text Word])) OR ("papillomavirus infections"[Text Word])) OR ("papillomavirus infection"[Text Word])) OR ("mycoplasma"[Text Word])) OR ("chlamydia"[Text Word])) OR ("reproductive tract infections"[Text Word])) OR ("reproductive tract infection"[Text Word])) OR ("trichomonas infections"[Text Word])) OR ("trichomonas infection"[Text Word])) OR ("chlamydia trachomatis"[Text Word])) OR ("neisseria gonorrhoeae"[Text Word])) OR ("mycoplasma genitalium"[Text Word])) OR ("trichomonas vaginalis"[Text Word])) OR ("trichomonas vaginitis"[Text Word])) OR ("mycoplasma hominis"[Text Word])) OR ("ureaplasma urealyticum"[Text Word])) OR ("ureaplasma"[Text Word])) OR ("sexually transmitted infections"[Text Word])) OR ("sexually transmitted infection"[Text Word])) OR ("trichomoniasis"[Text Word])) OR ("vaginosis"[Text Word])) OR ("ureaplasma parvum"[Text Word])) OR ("n gonorrhoeae"[Text Word])) OR ("viral sexually transmitted infection"[Text Word] OR "viral sexually transmitted infections"[Text Word])) OR (bacterial sexually transmitted disease[Text Word]))) AND (((((((("coinfection"[Text Word]) OR ("coinfections"[Text Word])) OR ("co infection"[Text Word])) OR ("co infections"[Text Word])) OR ("monoinfection"[Text Word])) OR ("monoinfections"[Text Word])) OR ("mono infection"[Text Word])) OR ("mono infections"[Text Word]))) AND (((((((((((((((((((((((("chorioamnionitis"[Text Word]) OR ("birth weight"[Text Word])) OR ("premature birth"[Text Word])) OR ("stillbirth"[Text Word])) OR ("adverse outcomes"[Text Word])) OR ("adverse outcome"[Text Word])) OR ("adverse pregnancy outcomes"[Text Word])) OR ("adverse pregnancy outcome"[Text Word])) OR ("birthweight"[Text Word])) OR ("macrosomia"[Text Word])) OR ("maternal outcomes"[Text Word])) OR ("maternal outcome"[Text Word])) OR ("outcomes"[Text Word])) OR ("outcome"[Text Word])) OR ("lower birthweight"[Text Word])) OR ("lower birth weight"[Text Word])) OR ("preterm"[Text Word])) OR ("adverse neonatal"[Text Word])) OR ("adverse maternal outcomes"[Text Word])) OR ("adverse maternal outcome"[Text Word])) OR ("preterm birth"[Text Word])) OR ("abortion"[Text Word])) OR ("pregnancy outcome"[Text Word])) OR ("pregnancy outcomes"[Text Word])))

**Supplemental Figure 1:** Funnel plot considering the five studies included in the prenatal delivery analysis

**Supplemental Figure 2:** Forest plot of preterm delivery (<37 weeks of gestational age) events according to the number of maternal non-viral reproductive tract infections, excluding studies representing more than 95% of the analysis weight (Felske et al). Odds ratios were determined with the Mantel-Haenszel random-effects method.

Abbreviations: OR, odds ratio; CI, confidence interval.

**Supplemental Figure 3:** Forest plot of delivery before 34 weeks of gestational age events according to the number of maternal non-viral reproductive tract infections. Odds ratios were determined with the Mantel-Haenszel random-effects method.

Abbreviations: OR, odds ratio; CI, confidence interval.

**Supplemental Figure 4:** Forest plot of preterm delivery (<37 weeks of gestational age) events comparing chlamydia mono-infection and chlamydia co-infection. Odds ratios were determined with the Mantel-Haenszel random-effects method.

Abbreviations: OR, odds ratio; CI, confidence interval.

**Supplemental Figure 5:** Forest plot of preterm delivery (<37 weeks of gestational age) events comparing gonorrhea mono-infection and gonorrhea co-infection. Odds ratios were determined with the Mantel-Haenszel random-effects method.

Abbreviations: OR, odds ratio; CI, confidence interval.

**Supplemental Figure 6:** Forest plot of preterm delivery (<37 weeks of gestational age) events comparing syphilis mono-infection and syphilis co-infection. Odds ratios were determined with the Mantel-Haenszel random-effects method.

Abbreviations: OR, odds ratio; CI, confidence interval.

**Supplemental Figure 7:** Forest plot of gestational hypertension events according to the number of maternal non-viral reproductive tract infections. Odds ratios were determined with the Mantel-Haenszel random-effects method.

Abbreviations: OR, odds ratio; CI, confidence interval.

**Supplemental Figure 8:** Forest plot of eclampsia or pre-eclampsia with severe features events according to the number of maternal non-viral reproductive tract infections. Odds ratios were determined with the Mantel-Haenszel random-effects method.

Abbreviations: OR, odds ratio; CI, confidence interval.

**Supplemental Figure 9:** Forest plot of gestational hypertension events comparing gonorrhea mono-infection and gonorrhea co-infection. Odds ratios were determined with the Mantel-Haenszel random-effects method.

Abbreviations: OR, odds ratio; CI, confidence interval.

**Supplemental Figure 10:** Forest plot of eclampsia or pre-eclampsia with severe features events comparing gonorrhea mono-infection and gonorrhea co-infection. Odds ratios were determined with the Mantel-Haenszel random-effects method.

Abbreviations: OR, odds ratio; CI, confidence interval.

**Supplemental Figure 11:** Forest plot of preterm premature rupture of membranes events according to the number of maternal non-viral reproductive tract infections. Odds ratios were determined with the Mantel-Haenszel random-effects method.

Abbreviations: OR, odds ratio; CI, confidence interval.

**Supplemental Figure 12:** Forest plot of preterm premature rupture of membranes events comparing gonorrhea mono-infection and gonorrhea co-infection. Odds ratios were determined with the Mantel-Haenszel random-effects method.

Abbreviations: OR, odds ratio; CI, confidence interval.

**Supplemental Figure 13:** Forest plot of spontaneous abortion or stillbirth events according to the number of maternal non-viral reproductive tract infections. Odds ratios were determined with the Mantel-Haenszel random-effects method.

Abbreviations: OR, odds ratio; CI, confidence interval.

**Supplemental Figure 14:** Forest plot of neonatal death events according to the number of maternal non-viral reproductive tract infections. Odds ratios were determined with the Mantel-Haenszel random-effects method.

Abbreviations: OR, odds ratio; CI, confidence interval.

**Supplemental Figure 15:** Forest plot of neonatal intensive care unit admission events according to the number of maternal non-viral reproductive tract infections. Odds ratios were determined with the Mantel-Haenszel random-effects method.

Abbreviations: OR, odds ratio; CI, confidence interval.

**Supplemental Figure 16:** Forest plot of chorioamnionitis or intraamniotic infection events comparing gonorrhea mono-infection and gonorrhea co-infection. Odds ratios were determined with the Mantel-Haenszel random-effects method.

Abbreviations: OR, odds ratio; CI, confidence interval.

**Supplemental Figure 17:** Forest plot of neonatal sepsis events according to the number of maternal non-viral reproductive tract infections. Odds ratios were determined with the Mantel-Haenszel random-effects method.

Abbreviations: OR, odds ratio; CI, confidence interval.

**Supplemental Figure 18:** Forest plot of birthweight <2500g events according to the number of maternal non-viral reproductive tract infections. Odds ratios were determined with the Mantel-Haenszel random-effects method.

Abbreviations: OR, odds ratio; CI, confidence interval.

**Supplemental Figure 19:** Forest plot of mean birthweight according to the number of maternal non-viral reproductive tract infections. Inverse variance was determined with the Mantel-Haenszel random-effects method.

Abbreviations: SD, standard deviation; CI, confidence interval.

**Supplemental Figure 20:** Forest plot of birthweight <2500g events comparing chlamydia mono-infection and chlamydia co-infection. Odds ratios were determined with the Mantel-Haenszel random-effects method.

Abbreviations: OR, odds ratio; CI, confidence interval.

**Supplemental Figure 21:** Forest plot of birthweight <2500g events comparing gonorrhea mono-infection and gonorrhea co-infection. Odds ratios were determined with the Mantel-Haenszel random-effects method.

Abbreviations: OR, odds ratio; CI, confidence interval.

**Supplemental Figure 22:** Forest plot of newborns small for gestational age according to the number of maternal non-viral reproductive tract infections. Odds ratios were determined with the Mantel-Haenszel random-effects method.

Abbreviations: OR, odds ratio; CI, confidence interval.

**Supplemental Table 1**: Results of analyses comparing outcomes between any mono-infection and any co-infection

| **Analysis** | **Number of studies** | **Pooled OR (95%CI)** | **Heterogeneity** | |
| --- | --- | --- | --- | --- |
|  |  |  | **I^2^ (%)** | **P value** |
| Preterm delivery (<37 weeks of gestation) | 5 | 0.83 (0.78–0.89) | 4 | 0.39 |
| Preterm delivery (<37 weeks of gestation) excluding studies representing more than 95% of the previous analysis weight (Felske et al.) | 4 | 1.01 (0.73–1.38) | 0 | 0.44 |
| Preterm delivery (<34 weeks of gestation) | 2 | 0.66 (0.36–1.22) | 19 | 0.27 |
| Gestational hypertension | 2 | 1.30 (0.72–2.33) | 42 | 0.19 |
| Eclampsia | 2 | 1.14 (0.73–1.78) | 0 | 0.39 |
| Preterm premature rupture of membranes | 2 | 0.89 (0.50–1.58) | 0 | 0.47 |
| Abortion/stillbirth | 2 | 0.60 (0.32–1.14) | 0 | 0.81 |
| Neonatal sepsis | 1 | 0.79 (0.17–3.70) | NA | NA |
| Birthweight <2,500g | 2 | 0.55 (0.19–1.57) | 88 | <0.01 |
| Small for gestational age | 2 | 0.85 (0.78–0.94) | 0 | 0.70 |
| Apgar <7 at five minutes | 3 | 0.53 (0.20–1.36) | 58 | 0.09 |
| Neonatal intensive care unit admission | 2 | 0.16 (0.03–0.86) | 0 | 0.41 |
| Chorioamnionitis | 3 | 0.76 (0.24–2.42) | 63 | 0.07 |
| Neonatal death | 2 | 0.17 (0.05–0.63) | 0 | 0.39 |

**Supplemental Table 2**: Results of analyses comparing outcomes between specific mono-infections and specific co-infections

| **Comparison** | **Analysis** | **Number of studies** | **Pooled OR (95%CI)** | **Heterogeneity** | |
| --- | --- | --- | --- | --- | --- |
|  |  |  |  | **I^2^ (%)** | **P value** |
| **Chlamydia co-infection vs. Chlamydia mono-infection** | Preterm delivery (<37 weeks of gestation) | 2 | 0.81 (0.75–0.87) | 0 | 0.59 |
|  | Birthweight <2,500g | 2 | 0.50 (0.20–1.29) | 84 | 0.01 |
| **Gonorrhea co-infection vs. Gonorrhea mono-infection** | Preterm delivery (<37 weeks of gestation) | 3 | 0.97 (0.88–1.06) | 0 | 0.77 |
|  | Gestational hypertension | 2 | 1.30 (0.74–2.30) | 39 | 0.20 |
|  | Eclampsia | 2 | 1.12 (0.61–2.05) | 0 | 0.35 |
|  | Preterm premature rupture of membranes | 2 | 0.83 (0.47–1.49) | 0 | 0.51 |
|  | Birthweight <2,500g | 2 | 0.72 (0.27–1.93) | 70 | 0.07 |
|  | Chorioamnionitis | 2 | 1.17 (0.50–2.73) | 0 | 0.82 |
| **Syphilis co-infection vs. Syphilis mono-infection** | Preterm delivery (<37 weeks of gestation) | 2 | 1.02 (0.87–1.19) | 0 | 0.94 |
